# Paradoxical Embolism Causing Myocardial Infarction in a COVID-19 Patient Presenting With Pulmonary Embolism

**DOI:** 10.7759/cureus.13975

**Published:** 2021-03-18

**Authors:** Mingxi D Yu, Nimit Desai, Thriveni Sanagala, Amir Darki

**Affiliations:** 1 Cardiology, Loyola University Medical Center, Chicago, USA

**Keywords:** covid19, pulmonary embolism, paradoxical embolism, myocardial infarction, clinical case report

## Abstract

The presence of myocardial injury in patients with severe acute respiratory syndrome coronavirus 2 (SARS-CoV2) infection is common. The cardiac complications of SARS-CoV2 infection are varied and distinguishing between them can be complicated.

A 55-year-old man with recent diagnosis of SARS-CoV2 infection presented with chest pain, syncope, and was found to have saddle pulmonary embolism (PE). Marked elevation in cardiac enzymes prompted a coronary angiogram which was normal. Cardiac MRI revealed late gadolinium enhancement (LGE) in the anterolateral wall consistent with myocardial infarction (MI). He was diagnosed with paradoxical embolism causing MI.

The differential for elevated cardiac enzymes is wide in patients with SARS-CoV2 infection. This case illustrates that sometimes multiple diagnoses exist, and that a high index of suspicion is required to continue work-up.

## Introduction

Myocardial injury and cardiac enzyme elevation in severe acute respiratory syndrome coronavirus 2 (SARS-CoV2) is common and has been associated with worse survival [1]. Unfortunately, a rise in cardiac enzymes has a wide differential, many of which can be seen among the cardiac complications of SARS-CoV2. We present a case of SARS-CoV2 related pulmonary embolism and a surprising diagnosis of myocardial infarction (MI) from a suspected paradoxical embolism. 

## Case presentation

**Figure 1:**
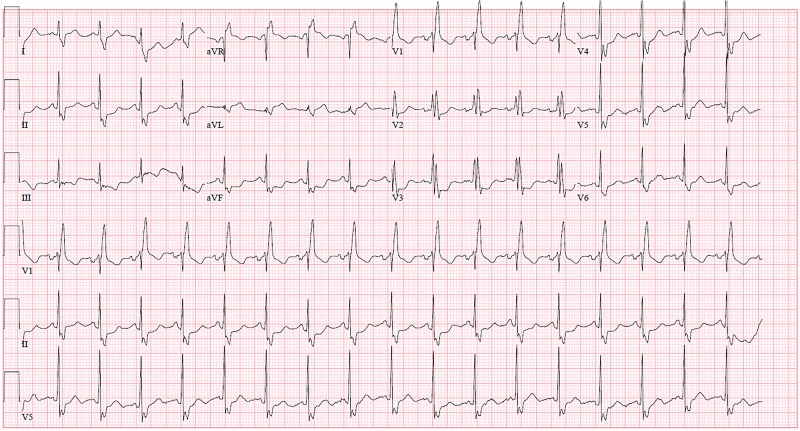
Presenting EKG. Admission electrocardiogram (EKG) showing sinus tachycardia, right bundle branch block (RBBB), and inferolateral ST-segment depressions

A 55-year-old man with a recent diagnosis of hypertension, diabetes, and SARS-CoV2 infection one week prior presented by ambulance with an episode of substernal chest pain and syncope at home. During his prior hospitalization for COVID-19, he was treated supportively and with prophylactic enoxaparin. At that time, a CT pulmonary embolism (PE) protocol and lower extremity dopplers were both negative. Ultimately, he was discharged home after three days without any anticoagulation. On arrival the patient was afebrile, with a heart rate of 108 bpm, blood pressure of 94/63 mmHg, and oxygen saturation of 92%. Per emergency medical services there was initial concern for ST-elevation MI in the field, however the arrival electrocardiogram (EKG) in the emergency room revealed sinus tachycardia, right bundle branch block (RBBB), and inferolateral ST-segment depressions (Figure 1).

**Figure 2:**
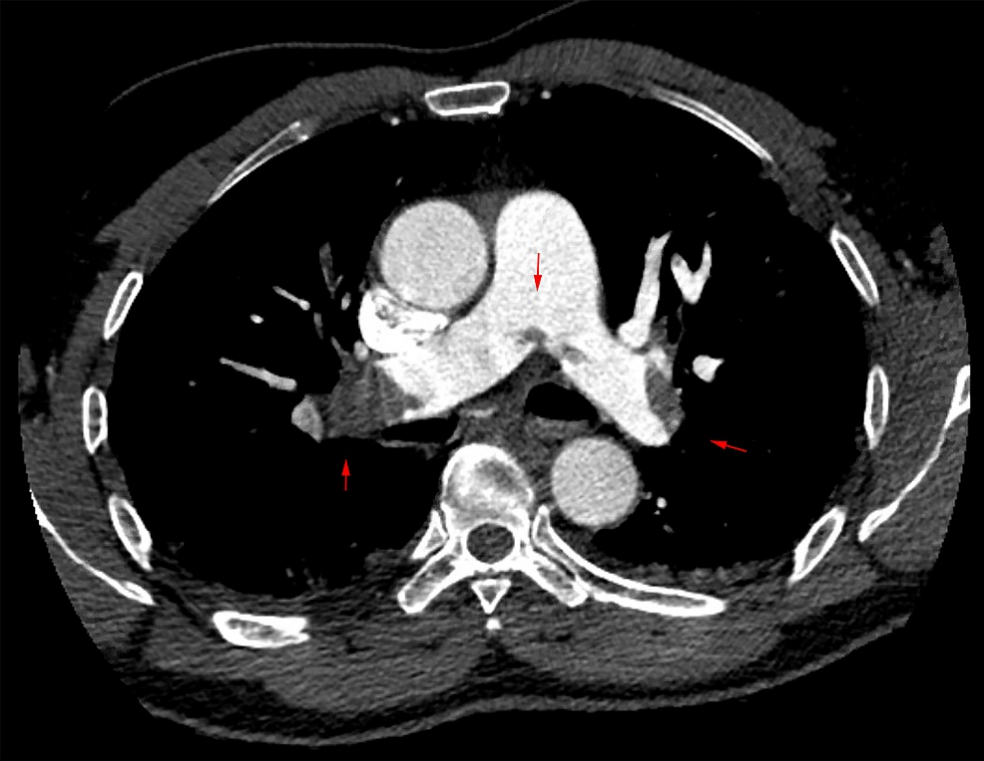
CT pulmonary angiogram. Presenting CT pulmonary embolism (PE) showing saddle pulmonary embolus in the axial view. Red arrows point to filling defects seen in the main pulmonary arteries and also at the bifurcation.

Laboratory values were significant for a D-Dimer of 38,598 ng/mL (Ref: <500 ng/mL), recent discharge D-Dimer 680 ng/mL, troponin 0.03 ng/mL (Ref: 0.00-0.02 ng/mL), brain natriuretic peptide (BNP) 40 pg/mL (Ref: 1-100 pg/mL), and lactate 1.3 mm/L (Ref: 0.9-1.7 mm/L). Due to having a history of COVID-19 and now hypoxia, a CT angiogram was done. The CT revealed a saddle PE with complete occlusion of the bilateral lobar arteries, right ventricular strain, and bilateral patchy ground-glass opacities (Figure 2).

He was treated with unfractionated heparin (UFH) and was transferred to the COVID-19 ICU for management of PE. After initiation of anticoagulation, oxygen support, and fluid resuscitation, the blood pressure improved and systemic thrombolysis was deferred.

Follow-up troponin obtained 13 hours after presentation was elevated to 72.25 ng/mL. At this time the patient denied any anginal symptoms and EKGs continued to show sinus tachycardia, RBBB, and inferolateral ST depressions. An echocardiogram showed a normal left ventricular systolic function, a positive bubble study after two beats (Video 1), and a pulmonary artery systolic pressure of 52 mmHg. 

**Video 1:** Echocardiogram with bubble study. Admission echocardiogram apical four-chamber view showing a normal left ventricular systolic function. With valsalva, bubbles appear on the left side of the heart after two beats from right sided opacification, indicating a positive bubble study and intracardiac shunt.

Due to the extensive degree of clot burden seen on CT as defined by a modified miller score of 22, and evidence of significant right ventricle (RV) strain; increased RV/left ventricle (LV) ratio 1.7, elevated lactate, reduced right ventricular outflow tract velocity time integral (RVOT VTI 7.9 cm (normal >10 cm) [2], the decision was made to escalate management of his PE to catheter directed thrombolysis (CDT) with alteplase. Upon completion of CDT, the patient’s hemodynamics improved (pulmonary artery, PA systolic pressure improved from 45 to 26 mmHg) on hospital day 1.

The troponin elevation was thought to be out of proportion to what was typically seen with acute PE. In the setting of SARS-CoV2 infection, we also considered acute myocarditis or acute coronary syndrome. Therefore, on hospital day two, the patient underwent coronary angiogram which revealed normal coronaries (Videos 2-3). 

**Video 2:** Coronary angiogram. Coronary angiogram in the right anterior oblique caudal projection showing normal left anterior descending artery (LAD) and circumflex arteries.

**Video 3:** Coronary angiogram. Coronary angiogram in the left anterior oblique projection showing a normal right coronary artery.

**Figure 3:**
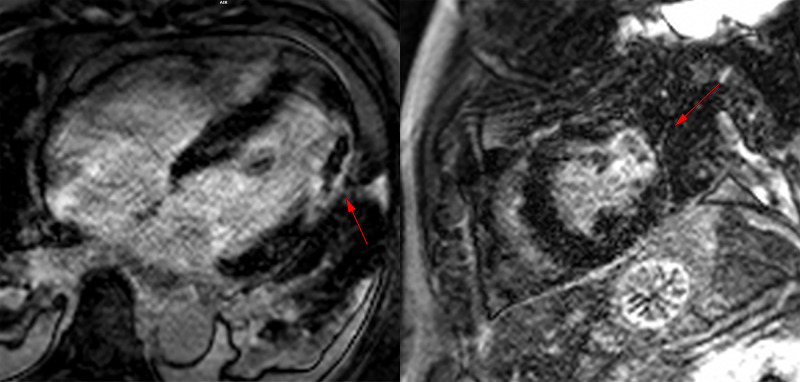
Cardiac MRI. Cardiac MRI with late gadolinium enhancement (LGE) images of the apical four chamber (left) and the short axis views (right). The LGE images showing transmural scar of the anterolateral wall as indicated by the red arrows.

A cardiac MRI was done to diagnose suspected myocarditis, but instead showed late gadolinium enhancement (LGE) in the mid-anterolateral wall with associated akinesis and a left ventricular ejection fraction of 50% (Figure 3). This pattern of LGE was consistent with a transmural scar and indicated that an infarct had occurred.

**Figure 4:**
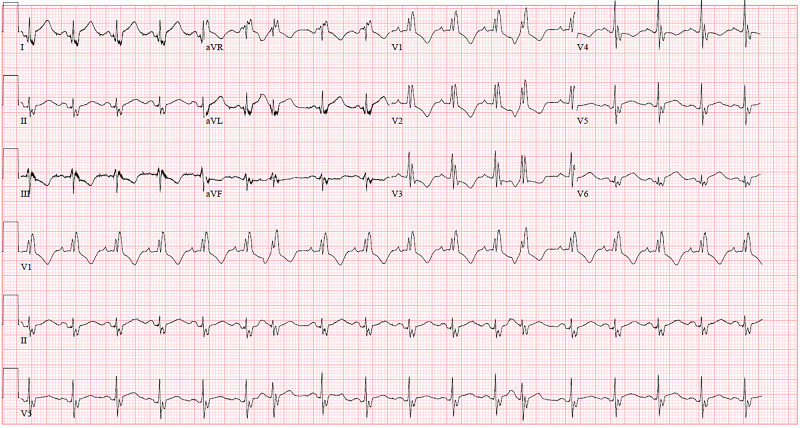
Discharge EKG. Discharge electrocardiogram (EKG) showing sinus tachycardia, right bundle branch block (RBBB), and a new lateral infarct.

The T2 mapping sequence did not show evidence of edema. Based on these findings he was diagnosed with an MI with a likely source of paradoxical coronary embolism via a patent foramen ovale (PFO). The patient continued to improve to the point where he required no supplemental oxygen and was ultimately discharged home on hospital day 7 with apixaban (Table 1). His discharge EKG had evidence of a new lateral infarct (Figure 4).

**Table 1:** Timeline of events. SARS-CoV2, severe acute respiratory syndrome coronavirus 2; PE, pulmonary embolism; PFO, patent foramen ovale; LGE, late gadolinium enhancement

Day -7	Patient was discharged from an outside hospital for a three-day admission with SARS-CoV2 infection.
Day 0	Admitted to ICU with PE (Figure 2) and was placed on heparin drip, initial troponin negative.
Day 0, 13 h	Follow up troponin markedly elevated to 72.25 ng/mL. No change in symptoms.
Day 1	Transthoracic echocardiogram showing PFO (Video 1). Underwent catheter directed thrombolysis.
Day 3	Remains persistently tachycardic with oxygen requirement. Underwent coronary angiogram revealing normal coronaries (Videos 2-3).
Day 5	Transferred out of the ICU.
Day 6	Cardiac MRI revealed LGE enhancement indicating transmural infarct (Figure 3).
Day 7	Patient was discharged home on a direct oral anticoagulant.

## Discussion

There is increasing evidence to suggest that the pathophysiology of COVID-19 is related to a combination of direct viral injury, maladaptive systemic inflammation, and hypercoagulability [3-4]. These pathologies are also potential mechanisms for the multitude of cardiac sequelae that have been observed, ranging from fulminant myocarditis to acute coronary syndrome. 

In this case, the differential for his constellation of chest pain and syncope was broad. Although his presentation was initially attributed to the PE noted on imaging, the rate and extent of troponin elevation prompted further investigation for additional etiologies. Myocardial injury is relatively common in critically ill patients with SARS-CoV2 infection with one series showing a prevalence of 19.7% [1]. The causes of troponin elevation with SARS-CoV2 infection are varied and include ischemia, stress cardiomyopathy, right heart strain, and myocarditis. Myocardial injury has been shown to be an independent risk factor for increased mortality, with the degree of troponin elevation positively correlating with worse outcomes [1]. Due to this strong association with mortality, it was deemed important to formally diagnose a cause for this marked elevation in troponin in an attempt to fully mitigate risk. The combination of normal coronary anatomy and the presence of MI on cardiac MRI was surprising. Given the lack of coronary disease or thrombus noted on cardiac catheterization, it is likely that there was a coronary embolus that recanalized with UFH therapy or with the CDT intended for treatment of the PE. Coronary embolism is a rare cause of MI with an estimated incidence of 2.9% in one 1,779 patient case series. The majority of these cases were attributed to atrial fibrillation (73%) [5], which our patient did not have. We could not exclude the possibility of a simultaneous coronary thrombosis as a cause for his infarction. Re-examination of the admission CT pulmonary angiogram did not show any obvious filling defects of the coronaries. It was felt that due to the presence of a large PE and also the markedly abnormal bubble study, a paradoxical embolism was more likely. A transesophageal echocardiogram and lower extremity dopplers were not performed.

In the era of COVID-19, it is not uncommon to see multiple pathologies present, in particular thrombotic complications in multiple territories. In retrospect, his initial presentation noted by EMS of STEMI may have reflected transient coronary clot in addition to his PE. The notion of Occam’s razor where one unifying diagnosis explains multiple findings, is perhaps less applicable to patients with SARS-CoV2 infection.

## Conclusions

The diagnosis of COVID-19 related cardiac sequelae is complicated as troponin elevation is a non-specific finding and can have multiple simultaneous explanations. A high of index of suspicion is required to diagnose MI in these patients, especially if another reasonable diagnosis has already been made.
